# Three exonic variants in the COL4A5 gene alter RNA splicing in a minigene assay

**DOI:** 10.1002/mgg3.2395

**Published:** 2024-02-23

**Authors:** Ran Zhang, Yanhua Lang, Xiaomeng Shi, Yiyin Zhang, Xuyan Liu, Fengjiao Pan, Dan Qiao, Xin Teng, Leping Shao

**Affiliations:** ^1^ Department of Nephrology the Affiliated Qingdao Municipal Hospital of Qingdao University Qingdao China; ^2^ Department of Materials the Affiliated Qingdao Municipal Hospital of Qingdao University Qingdao China; ^3^ Department of Ultrasound the Affiliated Qingdao Municipal Hospital of Qingdao University Qingdao China

**Keywords:** Alport syndrome, *COL4A5*, exon splicing, exonic variant, minigene analysis

## Abstract

**Background:**

X‐linked Alport syndrome (XLAS) is an inherited renal disease caused by rare variants of *COL4A5* on chromosome Xq22. Many studies have indicated that single nucleotide variants (SNVs) in exons can disrupt normal splicing process of the pre‐mRNA by altering various splicing regulatory signals. The male patients with XLAS have a strong genotype–phenotype correlation. Confirming the effect of variants on splicing can help to predict kidney prognosis. This study aimed to investigate whether single nucleotide substitutions, located within three bases at the 5′ end of the exons or internal position of the exons in *COL4A5* gene, cause aberrant splicing process.

**Methods:**

We analyzed 401 SNVs previously presumed missense and nonsense variants in *COL4A5* gene by bioinformatics programs and identified candidate variants that may affect the splicing of pre‐mRNA via minigene assays.

**Results:**

Our study indicated three of eight candidate variants induced complete or partial exon skipping. Variants c.2678G>C and c.2918G>A probably disturb classic splice sites leading to corresponding exon skipping. Variant c.3700C>T may disrupt splicing enhancer motifs accompanying with generation of splicing silencer sequences resulting in the skipping of exon 41.

**Conclusion:**

Our study revealed that two missense variants positioned the first nucleotides of the 5′ end of *COL4A5* exons and one internal exonic nonsense variant caused aberrant splicing. Importantly, this study emphasized the necessity of assessing the effects of SNVs at the mRNA level.

## Graphical abstract

Three exonic variants in the COL4A5 gene alter RNA splicing in a minigene assay. Two missense variants positioned the first nucleotides of the 5′ end of COL4A5 exons and one internal exonic nonsense variant caused aberrant splicing. Emphasized the necessity of assessing the effects of SNVs at the mRNA level.

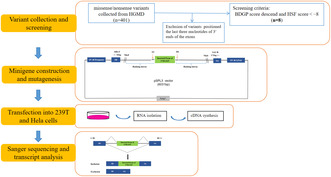

## 1 INTRODUCTION

Alport syndrome (AS), also known as hereditary nephritis, is a group of heterogeneous inherited disorders characterized by hematuria, proteinuria, and progressive kidney dysfunction and accompanied by hearing loss and ocular abnormalities and other less common extrarenal manifestation (Watson et al., 2023; Zhang & Ding, 2018). AS is caused by mutations in genes COL4A3, COL4A4 (2q36.3 both), and *COL4A5* (Xq22.3) encoding type IV collagen α3, α4, and α5 chains, respectively, which are the major components of the glomerular basement membrane (GBM) (Barker et al., 1990; Hudson et al., 2003; Longo et al., 2002).

The prevalence of Alport syndrome ranges from one in 5000 to one in 53,000 patients with kidney disease in different reports. The incidence of newly developed end‐stage renal disease (ESRD) is about 0.2%–0.5% in the adults and about 3%–12.9% in children (Hattori et al., 2015; Hicks et al., 2012; Mallett et al., 2014; Warady et al., 2020). The males are more likely to be symptomatic than females. According to its mode of inheritance, AS is grouped into X‐linked Alport syndrome (XLAS), autosomal recessive AS (ARAS), and autosomal dominant AS (ADAS). XLAS is caused by *COL4A5* gene mutations and accounts for 80% of patients with AS, while ARAS and ADAS are caused by homozygous or heterozygous mutations in COL4A3 or COL4A4 genes and account for 15% and 5% of patients with AS, respectively (Rheault & Kashtan, 2016).

The large‐scale data from published reports have analyzed the correlation between genotype and clinical picture in male XLAS patients (Bekheirnia et al., 2010; Gross, 2002; Jais et al., 2000; Nozu et al., 2019; Yamamura, Horinouchi, Nagano, et al., 2020). XLAS patients with nonsense mutation show the most severe phenotype, while who with missense mutations or splicing mutation present a mild phenotype or a moderate phenotype. In addition, in‐frame splicing variants appear significantly milder phenotypes than frameshift variants (Aoto et al., 2022; Horinouchi et al., 2018; Nozu et al., 2019; Yamamura et al., 2022; Yamamura, Horinouchi, Nagano, et al., 2020). So far, the total 1140 *COL4A5* gene variants have been described in the Human Gene Mutation Database (HGMD Professional 2023.1). There are 539 missense mutations/nonsense mutations (47.3%), 202 splice site mutations (17.7%), 174 small deletions (15.3%), 64 small insertions (5.6%), 11 small indels (1%), 136 gross deletions (11.9%), 6 gross insertions/duplications (0.5%), and 8 complex rearrangements (0.7%). It has been reported that exon single nucleotide variants (SNVs), which are often regarded as missense or nonsense mutations, even synonymous variants, may lead to abnormal splicing (Aoto et al., 2022; Horinouchi et al., 2020; Okada et al., 2023). Therefore, it is crucial to correctly identify whether these mutations have an impact on the splicing process.

The process of pre‐mRNA splicing removes introns from pre‐mRNA, and the remaining exons are combined to form mature mRNA. In the process of pre‐mRNA splicing, some specific sequences, such as the splice donor site (GU) and the splice acceptor site (AG) located in dinucleotides of both the 5′ and 3′ side of intron, respectively, the branch site and the polypyrimidine tract located upstream of the 3′ side of intron splice site, play an important role in determining the location of splicing (Yamamura et al., 2022). Furthermore, many splicing regulatory elements located on exons, such as exonic splicing enhancers (ESEs) and exonic splicing silencers (ESSs), also facilitate or repress the recognition of splice sites by spliceosome (Cartegni et al., 2002). It is well known that intron mutations can affect the splicing process by directly altering splicing sites (GU/AG, branch sites). Yet, many studies have revealed that mutations in exons, including missense, synonymous, or nonsense variants, can also result in abnormal pre‐mRNA splicing and activate cryptic splice sites by disrupting splicing signals (Baeza‐Centurion et al., 2020; Gonzalez‐Paredes et al., 2014).

At present, many studies have reported the effect of intron variation and synonymous variants in *COL4A5* gene on splicing (Boisson et al., 2023; Horinouchi et al., 2018, 2019, 2020). Nevertheless, the potential roles of the exonic SNVs located near exon–intron boundaries in causing aberrant splicing are overlooked. Related reports previously investigated the splicing effect of SNVs positioned the last nucleotide and 2nd or 3rd to the last nucleotide of exons in *COL4A5*, which revealed many variants caused aberrant splicing (Aoto et al., 2022; Okada et al., 2023). However, the effect of the SNVs positioned the first three nucleotides of the 5′ end of *COL4A5* exons and other exonic SNVs on splicing are still insufficient. Therefore, we assumed that these SNVs may also affect splicing. The aim of this study was to analyze the splicing effect of exonic variants in *COL4A5*, except SNVs positioned 2nd or 3rd to the last nucleotide of each exon.

## 2 MATERIALS AND METHODS

### 2.1 Variant nomenclature

DNA variant numbering was grounded in the complementary DNA (cDNA) sequence for *COL4A5* (NC_000023.11, NM_000495.5). The nomenclature of mutations was based on the guidelines of the Human Genome Variation Society (http://varnomen.hgvs.org), with c.1 representing the first nucleotide of the translation initiation codon.

### 2.2 Bioinformatics analyses and screening criteria

All reported missense and nonsense variants in *COL4A5* genes were collected from the Human Gene Mutation Database and ClinVar (July 2023). Each of these variants was analyzed to predict the effects on premRNA splicing through online bioinformatics software.

The analysis of BDGP (http://www.fruit fly.org) was performed to analyze the potential effects of variation on classic 5′ donor or 3′ acceptor consensus sites and/or to determine the generation and/or activation of novel sites. *COL4A5* variants, which located within 3 bases at the 5′ end of the exon and significantly reduced BDGP score, were selected to continue the analyses. The Human Splice Finder (version 3.1, available at http://www.umd.be/HSF3) was performed to investigate potential impact of missense or nonsense alterations on splicing regulatory sequences (ESEs broken and/or new ESSs creation) and identify the putative effect of variants on splicing regulatory motifs. The variants with HSF score (ESE/ESS motifs ratio) less than −8 were selected for further minigene splicing assays.

In this study, we selected *COL4A5* variants for experimental analyses according to the following criteria: (1) close to the 5′ ends of exons and (2) elimination of enhancers or creation of silencers (ESEs broken or new ESSs creation).

### 2.3 Minigene constructions and site‐directed mutagenesis

**FIGURE 1:**
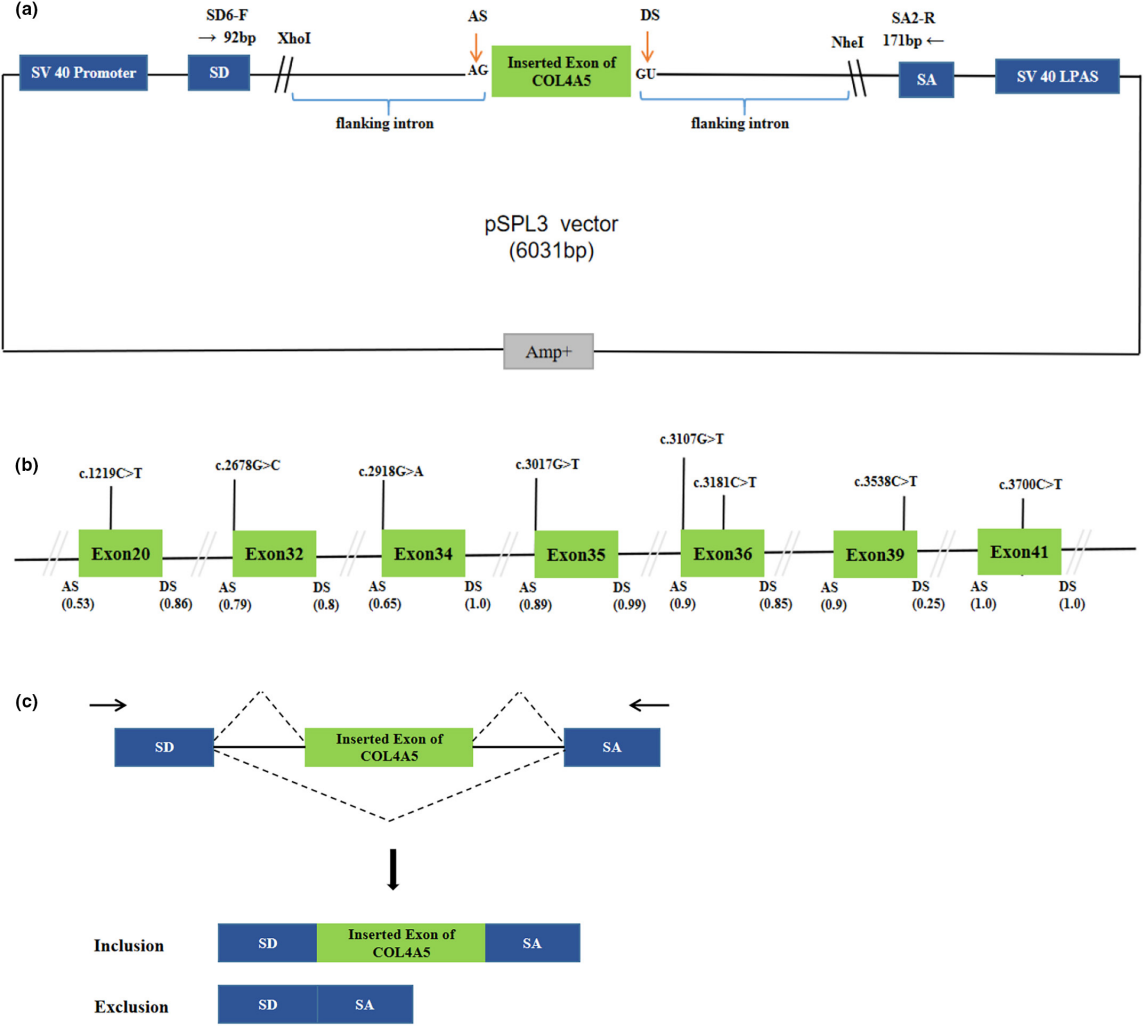
The schematic diagram of the minigene splicing assay constructed by pSPL3 exon trapping vector and position of 8 presumed missense and nonsense exonic variants selected in this study. (A) The pSPL3 vector includes two exons (SD and SA) and a functional intron. Transcription starts at the SV40 promoter and ends at the LPAS. The target exon containing partial flanking introns was inserted into pSPL3 vector via XhoI and NheI cloning sites to form the wild‐type or mutant plasmid. (B) Position of 8 variants in *COL4A5*. The green boxes and slashes between them represent the coding exons and introns sequences, respectively, and their sizes are out of proportion. The BDGP scores of donor and acceptor splice sites are represented in decimal, as shown at the bottom. (C) The transcripts with inclusion or exclusion of inserted exons of *COL4A5* produced by the hybrid minigene are schematically shown, and the arrows show the primers used to amplify. Dotted lines show the splice sites used in each case. LPAS, late poly(A) signal; AS, acceptor splice site; and DS, donor splice site.

This study was approved by the Ethics Committee of Qingdao Municipal Hospital affiliated with Qingdao University and obtained informed consent from the subjects. The genomic DNA from blood samples of healthy individuals was gained by GenElute Blood Genomic DNA Extraction Kit (Sigma, NA2010). The constructions of minigene have been described in the previous report (Zhang et al., 2021). The pSPL3 exon trapping vector was used in vitro minigene splicing assay, as shown in Figure 1A,C. The fragments with the wild‐type (WT) alleles consisting of target exons and shortened flanking introns of 50–200 nucleotides were amplified by PCRs and specific primers (Tables S1 and S3). These primers contain XhoI and NheI restriction sites (XhoI: CCGC^CTCGAG; NheI: CTAG^CTAGC), which facilitate cloning into the splicing vector pSPL3, and were designed by Primer Premier 5 and Primer‐Blast (http://www.ncbi.nlm.nih.gov/tools/primer‐blast). PCR products were purified by Gel Extraction Kit (CWBIO China). The purified PCR products and pSPL3 exon trapping vector were digested by enzymes XhoI and NheI, respectively. Then, PCR products were cloned into the exon trapping vector. The monoclonal colonies were screened and sequenced by forward primers, and the sequencing results are shown in Figure S1. SnapGene software was used for sequence alignment analysis. The plasmid DNA of positive monoclonal colonies was extracted using Pure Plasmid Mini Kit (Cwbio, China).

Mutagenesis primers were designed by SnapGene and Primer BLAST, as shown in Table S2. Variants of interest were introduced into *COL4A5* exons with QuikChange II Site Directed Mutagenesis Kit (Stratagene, La Jolla, CA, United States) according to the manufacturer's instruction. Primer extension and PCR amplification reactions are as follows: The first step is denaturation at 98°C for 10 s, followed by 30 cycles, denaturation at 98°C for 10 s, annealing at 62–55°C for 10 s, elongation at 72°C for 2 min, and finally extension at 72°C for 5 min. All constructed minigenes were confirmed through direct sequencing for ensuring obtained target variants (Figure S1).

### 2.4 Minigene splicing assay

Human embryonal kidney 293T (HEK293T) and Hela cells were cultured in DMEM with high glucose (4.5 g/L), supplemented with 10% fetal bovine serum, penicillin (100 U/L) and streptomycin (100 mg/L), and incubated at 37°C in a 5% CO_2_. One day before transfection, cells were seeded on 24‐well plate to grow to 70%–80% confluence in an antibiotic‐free medium. 1 μg plasmid DNA in each group of minigenes (empty pSPL3‐control, pSPL3‐WT, and pSPL3‐Mutation) was transfected to HEK239T and Hela cells using Lipofectamine 2000 (Invitrogen, United States) following the manufacturer's instructions.

After forty‐eight hours, total RNA was extracted with RNA‐easy Isolation Reagent (Vazyme Biotech Co., Ltd, China). First‐strand cDNA was synthesized from 1 ug of total RNA by RT‐PCR (reverse transcription PCR) using PrimeScript 1st Strand cDNA Synthesis kit (Takara, Japan) (Shao et al., 2018) under the instruction booklet of manufacturer. The PCR amplification reaction of cDNAs was executed with vector‐specific primers: SD6 (the forward primer: 5′‐TCTGAG TCACCTGGACAACC‐3′) and SA2 (the reverse primer: 5′‐ATCTCAGTGGTATTTGTGAGC‐3′). The PCR amplification reaction was performed as follows: in 30 μL volume, 3 μL of cDNA, 10 μL of 2 × Tap Plus MasterMix (Vazyme, China), 1 μL of each primer, and 15 μL of ddH2O. Thermal conditions were denaturation at 95°C for 3 min, 5 cycles of 95°C for 20 s, 62°C for 30 s, and 72°C for 30 s, 5 cycles of 95°C for 30 s, 60°C for 20 s, and 72°C for 30 s, 10 cycles of 95°C for 30 s, 58°C for 20 s, and 72°C for 30 s, 10 cycles of 95°C for 30 s, 55°C for 20 s, and 72°C for 30 s, and followed by a final elongation step at 72°C for 10 min. 1.5% agarose gel electrophoresis was used to resolve PCR products. The software ImageJ was used to quantify signal intensity of each band. The target DNA bands were cut out and purified using a Gel Extraction Kit (Cwbio, China). All transcripts were sequenced as previously described. The SnapGene software was used to compare DNA sequences with the reference *COL4A5* sequence published in GenBank. If the splicing pattern was different from the WT minigene in both HEK 293T and Hela cells, variation was considered to result in aberrant splicing, and the stability and reliability of the results were verified by three repeated experiments.

### 2.5 Statistical analysis

Quantification of the abnormal splicing percentage was densitometrically calculated as the percentage of exon exclusion (%) = (lower band/[lower band + upper band]) × 100. Statistical analysis was performed using SPSS software. The results were analyzed using the unpaired Student's *t*‐test by GraphPad Prism (Version 8.0.1; GraphPad Software). Error bars represent SEM (*n* = 3). *p* < 0.05 was considered statistically significant.

## 3 RESULTS

A total of 401 missense and nonsense variants compiled in the *COL4A5* database were analyzed using the bioinformatics software. We eliminated these variants positioned the last three nucleotides of 3′ ends of the exons in the *COL4A5* gene, which have been verified by minigene assay (Aoto et al., 2022; Okada et al., 2023). All screened mutations were analyzed with BDGP for splice site prediction and with HSF for ESE/ESS estimation algorithms in silico. We finally selected four variants within three bases of 5′ ends of the exons that have a weak 3′ splice site and four variants predicted to have an effect on splicing regulatory elements by HSF following the screening criteria (BDGP score descend and HSF score < −8). The enrolled variants were as follows: c.1219C>T, c.2678G>C, c.2918G>A, c.3017G>T, c.3107G>T, c.3181C>T, c.3538C>T, c.3700C>T, located in seven exons of the *COL4A5* gene, as shown in Table 1 and Figure 1B.

**TABLE 1:** Exonic variants in *COL4A5* selected from this study and the results of silico analyses.

Variant		Exon/length (bp)	Location in exon^a^	BDGP	HSF^c^	New ESS site^d^	ESE site broken^d^	Age range of ESRD	References
c.1219C>T	p.Gln407*	20/174	+54	NA	−9	5	4	18	Martin et al. (1998)
c.2678G>C	p.Gly893Ala	32/90	+1	3′AS: 0.79 → 0.17 (78.48%)^b^	NA			16–33	Mallett et al. (2017)
c.2918G>A	p.Gly973Asp	34/99	+1	3′AS: 0.65 → 0.34 (47.69%)^b^	NA			34	Morinière et al. (2014)
c.3017G>T	p.Gly1006Val	35/90	+1	3′AS: 0.89 → 0.49 (44.94%)^b^	NA			ND	Barker et al. (2001)
c.3107G>T	p.Gly1036Val	36/140	+1	3′AS: 0.9 → 0.49 (45.56%)^b^	NA			ND	Knebelmann et al. (1996)
c.3181C>T	p.Gln1061*	36/140	−66	NA	−9	1	9	21	Plant et al. (1999)
c.3538C>T	p.Gln1180*	39/99	−16	NA	−16	2	14	ND	Knebelmann et al. (1996)
c.3700C>T	p.Gln1234*	41/186	−91	NA	−9	7	3	42	Bekheirnia et al. (2010)

*Note*: *COL4A5* reference sequence: NC_000023.11, NM_000495.5.

Abbreviations: AS, acceptor splice sites; ESE, exonic splicing enhancer; ESS, exonic splicing silencer; NA, not applicable; ND, not determined.

^a^ Location of 8 variants: “+” indicates distance from the 5′ end of the exon, and “−” represents distance from the 3′ end of the exon.

^b^ Score changes with BDGP expressed in percentage.

^c^ ESE/ESS motifs ratio.

^d^ Values 0, 2, 3, 4, 5, 6, and 7 indicate the number of the splicing regulatory elements gained or disrupted.

**FIGURE 2:**
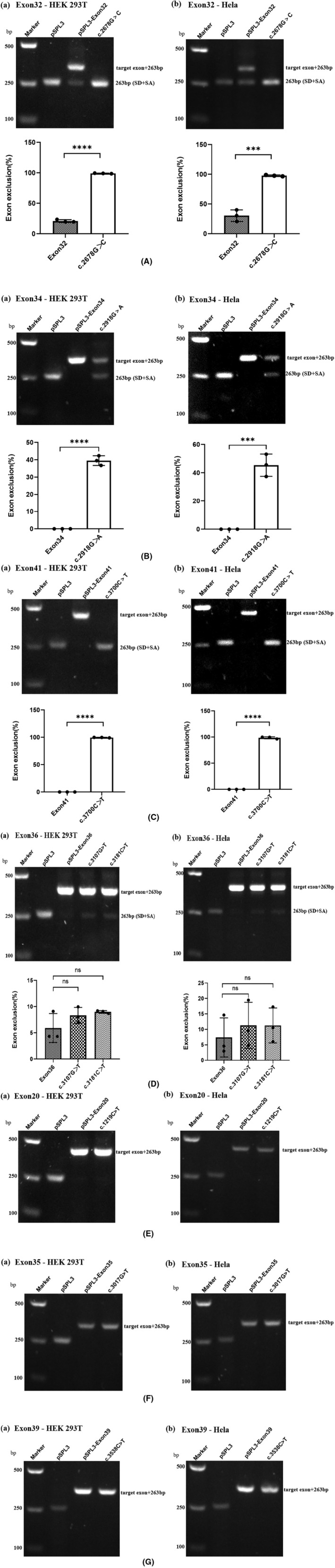
Agarose gel electrophoresis and statistical analysis of RT‐PCR expressed from the *COL4A5* minigene products in HEK 293T and Hela cells, respectively. The percentage of exon exclusion (%) = (lower band/[lower band + upper band]) × 100. Error bars represent SEM (*n* = 3). *p < 0.05; **p < 0.01; ***p < 0.001; ****p < 0.0001; ns: no significance; and unpaired Student's *t*‐test. A (a, b) Lane 1: marker; lane 2: pSPL3 (263 bp); lane 3: pSPL3‐Ex32 (353 and 263 bp); and lane 4: c.2678G>C (263 bp). B (a, b) Lane 1: marker; lane 2: pSPL3 (263 bp); lane 3: pSPL3‐Ex34 (362 bp); and lane 4: c.2918G>A (362 and 263 bp). C (a, b) Lane 1: marker; lane 2: pSPL3 (263 bp); lane 3: pSPL3‐Ex41 (449 bp); and lane 4: c.3700C>T (263 bp). D (a, b) Lane 1: marker; lane 2: pSPL3 (263 bp); lane 3: pSPL3‐Ex36 (403 and 263 bp); lane 4: c.3107G>T (403 and 263 bp); and lane 5: c.3181C>T (403 and 263 bp). E (a, b) Lane 1: marker; lane 2: pSPL3 (263 bp); lane 3: pSPL3‐Ex20 (437 bp); and lane 4: c.1219C>T (437 bp). F (a, b) Lane 1: marker; lane 2: pSPL3 (263 bp); lane 3: pSPL3‐Ex35 (353 bp); and lane 4: c.3017G>T (353 bp). G (a, b) Lane 1: marker; lane 2: pSPL3 (263 bp); lane 3: pSPL3‐Ex39 (362 bp); and lane 4: c.3538C>T (362 bp).

**FIGURE 3:**
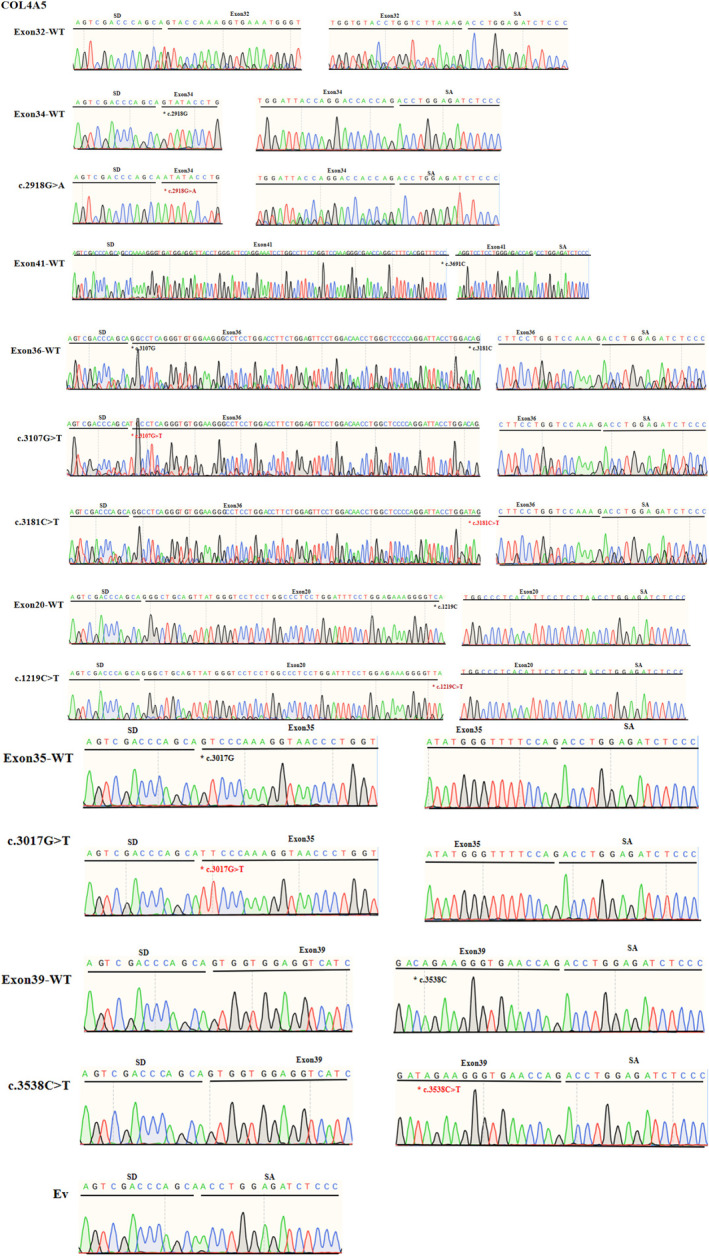
Sanger sequencing analysis figure. The sequencing results displayed that the larger fragment of each lane in Figure 2 included the target exon, SD and SA exons of the pSPL3 vector, while the smaller one included pSPL3 SD and SA exons (EV); * indicates the variant site.

We performed minigene splicing assays in vitro according to this bioinformatics data. The corresponding control minigenes were generated comprising *COL4A5* WT sequences of each exon (pSPL3 Ex20, pSPL3 Ex32, pSPL3 Ex34, pSPL3 Ex35, pSPL3 Ex36, pSPL3 Ex39, pSPL3 Ex41), respectively. All candidate variant minigenes were constructed through site‐directed mutagenesis using corresponding control minigenes as a template (Figure 1B). Results of minigene analysis indicated that some of them resulted in aberrant pre‐mRNA splicing in vitro (Figure 2), which were verified by sequencing analysis (Figure 3). Among eight candidates screened by BDGP and HSF, five variants (c.2678G>C, c.2918G>A, c.3700C>T, c.3107G>T, and c.3181C>T) resulted in complete or partial skipping of exons and three variants (c.1219C>T, c.3017G>T, and c.3538C>T) caused no exon skipping. Though two variants (c.3107G>T and c.3181C>T) led to partial skipping of exon 36, the WT minigene of exon 36 (pSPL3 Ex36) also expressed partial skipping of exon. There was no significant difference between them (Figure 2).

### 3.1 Missense variant c.2678G>C (p.Gly893Ala) led to complete skipping of exon 32

Variant c.2678G>C (p.Gly893Ala) is located at the first base of exon 32. This variation decreases the score of the 3′ acceptor splice site from 0.79 to 0.17 by BDGP analysis (Table 1). The results of RT‐PCR showed a sole product of 263 bp in mutant minigene, while there were two products, a larger band of 353 bp and a smaller band of 263 bp, in WT minigene (Figure 2A‐a,b). By sequencing analysis of all bands, it was confirmed that the larger fragment corresponds to correctly spliced exons (*COL4A5* exon 32 and flanked by exon SD and exon SA of the pSPL3 vector) and the smaller fragment corresponds to a transcript without exon 32 (Figure 3). Complete skipping of exon 32 will result in an in‐frame deletion alteration (from 893rd codon to 922nd codon, corresponding to 30 amino acids). Therefore, variant c.2678G>C (p.Gly893Ala) abrogates the acceptor splice site and causes complete exon 32 skipping.

### 3.2 Missense variant c.2918G>A (p. Gly973Asp) resulted in partial skipping of exon 34

The variant c.2918G>A (p. Gly973Asp) affected the G nucleotide at position 1 of *COL4A5* exon 34, which is closely located at downstream of classical splice site AG dinucleotide. Bioinformatics predictions from BDGP showed that the variants reduced the score of the 3′ acceptor splice site from 0.65 to 0.34 (Table 1). We examined the experimental effect of variant c.2918G>A using the WT (pSPL3‐Exon 34) and mutant minigene. The minigene assay result identified that the WT and mutant minigenes generated different cDNA products, respectively. The WT lane just demonstrated one unique fragment of 362 bp that contains exon 34, SD and SA of the pSPL3, whereas mutant lane showed two different fragments of 362 bp corresponding to a transcript with exon 34 carrying variant c.2918G>A and 263 bp corresponding to the lack of exon 34 of mRNA (Figure 2B‐a). So, variant c.2918G>A (p. Gly973Asp) disturbed the 3′ AS and caused partial skipping of exon 34. Meanwhile, this result was confirmed by sequencing analysis. The partial skipping of 99‐bp exon 34 will lead to a 33‐aa in‐frame deletion.

### 3.3 Nonsense variant c.3700C>T (p. Gln1234*) prevented incorporation of exon 41 into the mature mRNA

Nonsense variant c.3700C>T (p. Gln1234*) located at internal position of exon 41 alters a CAG codon for Glu to a premature TAG stop codon. In silico analysis, this variant was predicted to make a significant alteration of ESE/ESS motifs ratio (−9) by HSF 3.1 software, not only resulting in broking three ESEs but also creating seven new ESSs (Table 1). RT‐PCR analysis results of minigenes indicated that the mutant and WT minigenes generated different products (Figure 2C‐a,b). The WT minigene produced a band of 449 bp, whereas the mutant minigene generated a unique product of 263 bp. The results were further confirmed by sequencing analysis (Figure 3), which showed that the larger band was the transcript containing exon 41 and the smaller one was the transcript excluding exon 41. Therefore, we consider that the pathogenicity of this variant was caused by in‐frame deletion with loss of 62‐aa rather than nonsense mutation.

### 3.4 Variant c.3107G>T (p. Gly1036Val) and c.3181C>T (p. Gln1061*) produced the exon 36‐excluded transcript

Missense variant c.3107G>T (p. Gly1036Val), altered G at the first nucleotide of exon 36 to T, was predicted to reduce the score of the acceptor splice site from 0.9 to 0.49 with BDGP (Table 1). Nonsense variant c.3181C>T (p. Gln1061*), caused by substitution of the 66th nucleotide of the 3′ end of the exon 41, was predicted to affect pre‐mRNA splicing with an HSF score of −9, along with broking nine ESEs and creating one new ESS (Table 1). However, direct sequencing results of the minigene assays showed that there were two bands, among which 403 bp represented transcript of exon 41 with wild‐type, c.3107G>T and c.3181C>T, respectively, as well as bands of 263 bp only contained pSPL3 exons (EV) (Figure 3). These bands of 403 and 263 bp were both detected in the WT and the mutant lane (Figure 2D‐a,b). Even so, these two mutants (c.3107G>T and c.3181C>T) resulted in partial exon 36 skipping and increased the amounts of the exon‐excluded transcripts compared with the WT minigene, and there was no significant difference (Figure 2D).

### 3.5 Variants in exon 20, exon 35, and exon 39 did not alter splicing of pre‐mRNA

Nonsense variant c.1219C>T (p. Gln407*) was located at the 54th nucleotide position from the 5′ end of exon 20. This variation was predicted to disrupt four ESE sites and generate five new ESS sites using HSF 3.1. Missense variant c.3017G>T (p. Gly1006Val), located at the first nucleotide position of exon 35, was predicted to alter the score of the acceptor splice site from 0.89 to 0.49 with BDGP (Table 1). Nonsense variant c.3538C>T (p. Gln1180*) was identified at internal 16th nucleotide position from the 3′ end of exon 39, which was analyzed by HSF 3.1 to break fourteen ESEs and create two novel ESSs. However, the RT‐PCR products of mutant minigenes were the same as those generated from WT minigenes (Figure 2E–G). These were further confirmed by sequencing analysis (Figure 3). Thus, these variants did not influence pre‐mRNA splicing.

## 4 DISCUSSION

At present, an increasing number of SNVs were verified to affect RNA splicing. About 8.6% human pathogenic variants reported in HGMD (released in 2021.4) have been found to influence pre‐mRNA splicing, and the reported number of splicing variants is likely underestimated. In addition, nearly 25% of known missense and nonsense variants alter splicing of exons (Sterne‐Weiler et al., 2011). There are many variants in *COL4A5* gene previously considered as missense and nonsense mutations. Moreover, the male patients with XLAS have a strong genotype–phenotype correlation. The mean age at onset of ESRD differed significantly among mutation categories: missense 37.5 years, splice site 29.0 years, and truncating 24.0 years (Bekheirnia et al., 2010). So, transcript analysis is necessary to reveal the effects of exonic SNVs in the *COL4A5* gene on potential splicing and understand kidney prognosis correctly.

It is well known that the RNA from patients with XLAS is an optimal experimental sample used to identify potential splicing variants. Regrettably, RNA is hard to obtain because of its instability and low content in peripheral blood. In the absence of RNA samples, minigene splicing analysis has become an effective alternative to evaluate the effect of SNVs on pre‐mRNA splicing, which has been validated among different diseases in our previous studies (Shi et al., 2023; Wang et al., 2020; Xin et al., 2022; Zhang et al., 2018, 2021; Zhao et al., 2016). In this study, pSPL3 minigenes were constructed and transfected into cultured HEK 293T and Hela cells, respectively. As a result, we revealed that two missense variants positioned the first nucleotides of the 5′ end of *COL4A5* exons and one internal exonic nonsense variant caused aberrant splicing. Previous reports showed that 17 out of 20 SNVs at the end of exons and 6 out of 8 SNVs positioned 2nd or 3rd to the last nucleotide of exons in *COL4A5* affected splicing (Aoto et al., 2022; Okada et al., 2023). Our findings complement their research.

The canonical splice signals and regulatory elements in the exonic or intronic sequences affected the diversity of gene splicing together. Therefore, we chose bioinformatics tools BDGP and HSF to predict the effect of missense and nonsense variants of *COL4A5* gene on splicing, empirically. Variants, near to classical splice sites, could result in skipping of the corresponding exon by inducing a significantly decreasing strength of splice site, which was confirmed by the minigene analysis of variants c.2678G>C and c.2918G>A in this study. In addition, variants in the exon, affected splicing regulatory sequences, led to the corresponding exon skipping, which was also revealed by the minigene analysis of variant c.3700C>T in our study.

It was reported that variants c.2678G>C (p.Gly893Ala) and c.2918G>A (p. Gly973Asp) were identified as missense variants (Mallett et al., 2017; Morinière et al., 2014). Bioinformatics analyses indicated these two variants decreased the recognition strength of the authentic acceptor splice of intron 31 and intron 33, respectively. Furthermore, the result of minigene analysis showed that variant c.2678G>C disturbed the normal splicing, causing complete skipping of exon 32. Subsequently, the connection between exons 31 and 33 would result in a lack of 30‐aa located in collagenous domain of type IV collagen α‐5 chain, which affected the formation of type IV collagen α‐5 chain. The age range for patients with variant c.2678G>C to ESRD was 16 to 33 years (Bekheirnia et al., 2010). For another mutation c.2918G>A, minigene analysis indicated that it weakened recognition of the 3′ splice site of intron 33, resulting in partial skipping of exon 34 and product lacking of 33 amino acids located in collagenous domain of type IV collagen α‐5 chain. The age for patient with variant c.2918G>A to ESRD was 34 years (Morinière et al., 2014). The relatively severe phenotypes also imply that these variants may be serious mutation types.

Bekheirnia had reported that variant c.3700C>T (p. Gln1234*) was identified as nonsense variants (Bekheirnia et al., 2010). According to the assessment of HSF, this variant affected related ESE and ESS motifs. The results of our minigene assays indicated that variant c.3700C>T produced transcript lacking the entire exon 41. Many regulatory elements in the exon, including ESEs and ESSs, promote or inhibit the identification of surrounding splice sites through recruiting diverse protein factors (Shao et al., 2018). In this study, we supposed that variant c.3700C>T may cause significant reduction in the proportion of ESEs/ESSs by destroying ESEs and generating ESSs. Consequently, the strength of identifying splice site is prominently decreased. The p.Gln1234* is located in collagenous domain of type IV collagen α‐5 chain. Nonsense mutations lead to the formation of truncated peptide chains and further cause the inability of type IV collagen α‐5 chain, accompanying with severe phenotype. However, the patient with variant c.3700C>T developed ESRD at age 42 (Bekheirnia et al., 2010), which was inconsistent with the severe phenotype of nonsense mutation. So, the variant c.3700C>T is virtually an in‐frame deletion (loss of 62 amino acids), which expressed milder phenotype, rather than a nonsense mutation. Additionally, c.3700C>T causes complete exon 41 skipping and the transcript excluding exon 41 does not introduce the premature termination codon, which will escape nonsense‐mediated RNA decay (NMD). This may be also a reason for the relatively mild phenotype of this mutation.

However, bioinformatics software and minigene assays also have the limitation in detecting and simulating splicing patterns compared to the situation in vivo. Variants c.1219C>T, c.3017G>T, and c.3538C>T, predicted by BDGP and HSF to have a significant impact on splicing, did not cause corresponding exon skipping. Although variants c.3107G>T and c.3181C>T produced exon 36‐excluded transcripts, no significant difference was found between mutant and WT minigenes. The inconsistency between experimental and predicted results was frequently reported in other studies (Aoto et al., 2022; Okada et al., 2023; Shi et al., 2023; Xin et al., 2022). There may be many reasons for this phenomenon, including the defects of predict software, the differential expression of splicing factors in vitro, sequence context in regulating splicing (Lin et al., 2021), etc. In addition, the shortcomings of this study are that we did not introduce these variants in cDNA based on prediction of single nucleotide substitution and the corresponding exon exclusion and meanwhile did not test for structure and function of type IV collagen α‐5 chain formed by different transcripts, which require further investigation.

It is worth noting that some presumed nonsense variants were sorted and reclassified as splicing variants, which was very helpful in phenotype/genotype association study and developing novel targeted gene therapy. An emerging therapeutic approach, called “exon skipping therapy,” was developed using single‐stranded antisense oligonucleotides (ASOs). Up to now, ASO therapeutics by targeting exon skipping have demonstrated promising therapeutic effects for various inherited diseases, such as eteplirsen, golodirsen, viltolarsen, and casimersen for Duchenne muscular dystrophy (Iftikhar et al., 2021; Migliorati et al., 2022). In XLAS, ASO therapy targeting variant c.1411C>T (p. Gln471*) in exon 21 of COL4A5 gene achieved the skipping of exon 21, which was verified to significantly improve the clinical phenotypes by mouse model of AS. This result suggested that exon skipping may represent a promising therapeutic approach for severe male XLAS cases (Yamamura, Horinouchi, Adachi, et al., 2020). In addition, there are also some studies about ASO therapeutics by targeting exon restoration, including nusinersen for spinal muscular atrophy, oligonucleotide‐induced alternative splicing of serotonin 2C receptor (Claborn et al., 2019; Zhang et al., 2016). Thus, we propose that more attention should be paid to the impact on splicing regulation of identified variants. The research and application of ASOs in urinary system will be worth looking forward.

## 5 CONCLUSION

Our results revealed that two missense variants positioned the first nucleotides of the 5′ end of *COL4A5* exons and one internal exonic nonsense variant caused aberrant splicing. Variants c.2678G>C and c.2918G>A probably disturb 3′ acceptor splice site leading to exon skipping. Variant c.3700C>T may disrupt splicing enhancer motifs and generate splicing silencer sequences resulting in skipping of exon 41. Furthermore, this study emphasized the necessity of assessing the effects of SNVs on accurately predicting mutational effect and helping to evaluate prognosis of XLAS at the mRNA level.

## AUTHOR CONTRIBUTIONS

Ran Zhang, Yanhua Lang, Leping Shao, and Xin Teng conceived and designed and performed the experiments. Ran Zhang, Xiaomeng Shi, Yiyin Zhang, Fengjiao Pan, and Dan Qiao performed the experiments. Ran Zhang and Xuyan Liu contributed to the data analysis. Ran Zhang wrote the manuscript. Xin Teng and Leping Shao revised the manuscript. All authors had read and approved the final manuscript.

## FUNDING INFORMATION

This study was funded by the National Natural Science Foundation of China (No. 82170717).

## CONFLICT OF INTEREST STATEMENT

The authors declare that they have no conflict of interest.

## ETHICS STATEMENT

The study was approved by the ethics committee of the Affiliated Qingdao Municipal Hospital of Qingdao University (No. 2018‐028). Informed consent was obtained from all participants included in this study.

## Supporting information


Data S1.

## Data Availability

The datasets generated during the current study can be found in the Human Gene Mutation Database (http://www.hgmd.org) and ClinVar (https://www.ncbi.nlm.nih.gov/clinvar/).
