# Supplementary material for: Three exonic variants in the COL4A5 gene alter RNA splicing in a minigene assay
Source: Mol Genet Genomic Med. 2024 Feb 23;12(2):e2395. doi: 10.1002/mgg3.2395 (PMC10891438; doi:10.1002/mgg3.2395)

**Table S1** Primer sequences for PCR amplification of exons in COL4A5 from this study

| **Exon-Primer Sequences (5’-3’) Product size (bp)** |
| --- |
| PSPL3-COL4A5 Exon20-F CCGCTCGAGGATTGTCAAAGGATGGGG 563  PSPL3-COL4A5 Exon20-R CTAGCTAGCGCAGAAGCAGTGGTAGAA  PSPL3-COL4A5 Exon32-F CCGCTCGAGGAATCAACCCTAGTTCCA 543  PSPL3-COL4A5 Exon32-R CTAGCTAGCGGTATCCAAACTAAGCAC  PSPL3-COL4A5 Exon34-F CCGCTCGAGCCAAAATAGGAAAGTGAT 576  PSPL3-COL4A5 Exon34-R CTAGCTAGCATACCCTGCTGCCACAAA  PSPL3-COL4A5 Exon35-F CCGCTCGAGTAGGGAAAGAAACAAGGT 487  PSPL3-COL4A5 Exon35-R CTAGCTAGCGAACACTACCCACTGATT  PSPL3-COL4A5 Exon36-F CCGCTCGAGTCAGTGGGTAGTGTTCTC 614  PSPL3-COL4A5 Exon36-R CTAGCTAGCGCCACAAACCCTAAAACA  PSPL3-COL4A5 Exon39-F CCGCTCGAGATTCTCCCCACCACCCTT 487  PSPL3-COL4A5 Exon39-R CTAGCTAGCTAGGAAAAGGGGAAAGTG  PSPL3-COL4A5 Exon41-F CCGCTCGAGTTTGGGATTTGGTAGGAG 515  PSPL3-COL4A5 Exon41-R CTAGCTAGCATGGAAATAGATGGAGTTAG |

**Table S2**  Mutagenesis primers sequences for exonic variants in COL4A5 from this study

| **Variant-Primer Sequences (5’-3’)** |
| --- |
| pSPL3-COL4A5-Exon20-c.1219C＞T -F GAAAGGGGTTAGAAAGGTGA  pSPL3-COL4A5-Exon20-c.1219C＞T -R TCACCTTTCTAACCCCTTTC  pSPL3-COL4A5-Exon32-c.2678G＞C -F CTTCAAAGCTACCAAAGGTG  pSPL3-COL4A5-Exon32-c.2678G＞C -R CACCTTTGGTAGCTTTGAAG  pSPL3-COL4A5-Exon34-c.2918G＞A -F CTTGGAAGATATACCTGGAG  pSPL3-COL4A5-Exon34-c.2918G＞A -R CTCCAGGTATATCTTCCAAG  pSPL3-COL4A5-Exon35-c.3017G＞T -F CCTTTCTAGTTCCCAAAGGT  pSPL3-COL4A5-Exon35-c.3017G＞T -R ACCTTTGGGAACTAGAAAGG  pSPL3-COL4A5-Exon36-c.3107G＞T -F TTTTCAACAGTGCCTCAGGGT  pSPL3-COL4A5-Exon36-c.3107G＞T -R ACCCTGAGGCACTGTTGAAAA  pSPL3-COL4A5-Exon36-c.3181C＞T -F ATTACCTGGATAGAAAGGCG  pSPL3-COL4A5-Exon36-c.3181C＞T -R CGCCTTTCTATCCAGGTAAT  pSPL3-COL4A5-Exon39-c.3538C＞T -F ACCAGCTGGATAGAAGGGTGAA  pSPL3-COL4A5-Exon39-c.3538C＞T -R TTCACCCTTCTATCCAGCTGGT  pSPL3-COL4A5-Exon41-c.3700C＞T -F TTTCCCTGGTGTGTAGGGTC  pSPL3-COL4A5-Exon41-c.3700C＞T -R GACCCTACACACCAGGGAAA |

**Table S3**  The sequences of wild-type and mutant exons with flanking introns COL4A5 from this study

| **Target Exon** | **Sequences(5’→3’)** |
| --- | --- |
| **COL4A5-Exon20** | gattgtcaaaggatggggttactagtaatttccaaaagtagtaaacctactttaaattattatttcctaccctcaaactgttacttacattctttgaaacttttcaacctttttttcagacttttgagtctcatagaaaaaaaataacataaccagaattatatgttaaaggaagatcttatcattatctaatgtctcaatgagaactaaagtaacatctctaagatgaaatcattttgatcacttttttgaatcttAGgggctgcagttatgggtcctcctggccctcctggatttcctggagaaaggggtcagaaaggtgatgaaggaccacctggaatttccattcctggacctcctggacttgacggacagcctggggctcctgggcttccagggcctcctggccctgctggccctcacattcctcctaGTaagctatatttttctcctattaagttctatttttgtttttgtttattttgtttataagtaactctagctaaaggttggcctcatttgttgcgtttctgatatctaagaaattctaccactgcttctgc |
| **COL4A5-Exon20 with c.1219C>T** | gattgtcaaaggatggggttactagtaatttccaaaagtagtaaacctactttaaattattatttcctaccctcaaactgttacttacattctttgaaacttttcaacctttttttcagacttttgagtctcatagaaaaaaaataacataaccagaattatatgttaaaggaagatcttatcattatctaatgtctcaatgagaactaaagtaacatctctaagatgaaatcattttgatcacttttttgaatcttAGgggctgcagttatgggtcctcctggccctcctggatttcctggagaaaggggttagaaaggtgatgaaggaccacctggaatttccattcctggacctcctggacttgacggacagcctggggctcctgggcttccagggcctcctggccctgctggccctcacattcctcctaGTaagctatatttttctcctattaagttctatttttgtttttgtttattttgtttataagtaactctagctaaaggttggcctcatttgttgcgtttctgatatctaagaaattctaccactgcttctgc |
| **COL4A5-Exon32** | gaatcaaccctagttccaggtgagggttttatagtagattgtacatactgggacatatggttcactggggggccatctttagagattagctaccagactgagtctccttattcatgggtatcagcagtagtgggcactatggtcaaaaagtctaaaacttaacagtgccttacgtccaaccctcaatagttttctggttgacatcttaaaacttactttttatgttccctaagtcaaagaaaggcaaacattacttattgatattcttcaaAGgtaccaaaggtgaaatgggtatgatgggacctccaggcccaccaggacctttgggaattcctggcaggagtggtgtacctggtcttaaagGTaataatcaaggtttgctgccagacgtatgtgagagggaaaattaaatatagctttatgtcagtacagaatatttttgttgactgttttaaaatgagcaatggttacttgtgtttctatgtaacatggcatttaaataggtcttgttttcaatttggttacgtgcttagtttggatacc |
| **COL4A5-Exon32 with c.2678G>C** | gaatcaaccctagttccaggtgagggttttatagtagattgtacatactgggacatatggttcactggggggccatctttagagattagctaccagactgagtctccttattcatgggtatcagcagtagtgggcactatggtcaaaaagtctaaaacttaacagtgccttacgtccaaccctcaatagttttctggttgacatcttaaaacttactttttatgttccctaagtcaaagaaaggcaaacattacttattgatattcttcaaAGctaccaaaggtgaaatgggtatgatgggacctccaggcccaccaggacctttgggaattcctggcaggagtggtgtacctggtcttaaagGTaataatcaaggtttgctgccagacgtatgtgagagggaaaattaaatatagctttatgtcagtacagaatatttttgttgactgttttaaaatgagcaatggttacttgtgtttctatgtaacatggcatttaaataggtcttgttttcaatttggttacgtgcttagtttggatacc |
| **COL4A5-Exon34** | ccaaaataggaaagtgatttcactactgagtgctctgaatttcttgatggtaggtaactaaaaagtgaaggattttcgtgtgagtccagtgctaatagctcatactatatcagaatatcaccagttcctctaattcacttatagtttaacacttgagtagcttgctttgccaaagttatttcatggatgaataatatcatcctaacttgcctcttctactcattcttggaAGgtatacctggagtttcagggccaaaaggttatcagggtttgcctggagacccagggcaacctggactgagtggacaacctggattaccaggaccaccagGTaagtgtgataggccatttgtagcaattgcttagctgacactgaattctggataaataattatgtgtttgtcatgtttgaagatgctgtggatttgatcatggtaataagcttgggtagctactgaaatatggagctcactttcggcaaaaatattgccctttggctctagttttagcagtccagattttataggatattttgccttgattattcagtttcttctttttgtggcagcagggtat |
| **COL4A5-Exon34 with c.2918G>A** | ccaaaataggaaagtgatttcactactgagtgctctgaatttcttgatggtaggtaactaaaaagtgaaggattttcgtgtgagtccagtgctaatagctcatactatatcagaatatcaccagttcctctaattcacttatagtttaacacttgagtagcttgctttgccaaagttatttcatggatgaataatatcatcctaacttgcctcttctactcattcttggaAGatatacctggagtttcagggccaaaaggttatcagggtttgcctggagacccagggcaacctggactgagtggacaacctggattaccaggaccaccagGTaagtgtgataggccatttgtagcaattgcttagctgacactgaattctggataaataattatgtgtttgtcatgtttgaagatgctgtggatttgatcatggtaataagcttgggtagctactgaaatatggagctcactttcggcaaaaatattgccctttggctctagttttagcagtccagattttataggatattttgccttgattattcagtttcttctttttgtggcagcagggtat |
| **COL4A5-Exon35** | tagggaaagaaacaaggtgttctggtaactttttaatttttttttaaagttatattaggagacctctattactatggttatattgtaatatgttcccaaggagatgttttatgttgggttctatctgtggaccttaatcatatattcagatttaatccatctaatcccatgtttgcattttaatgactatccattcccatgaaaccagacaaccccaatattgctacattgtcttaattttaccaatttgacctttctAGgtcccaaaggtaaccctggtctccctggacagccaggtcttataggacctcctggacttaaaggaaccatcggtgatatgggttttccagGTgagtgatgaaaatcttccaaatatttagtcccattaatgaaaggtggttcaatatctctttttttgtcagaaaagaggctggtgttgatagaatcagactgaaacgatatctgaggtaatcagtgggtagtgttc |
| **COL4A5-Exon35 with c.3017G>T** | tagggaaagaaacaaggtgttctggtaactttttaatttttttttaaagttatattaggagacctctattactatggttatattgtaatatgttcccaaggagatgttttatgttgggttctatctgtggaccttaatcatatattcagatttaatccatctaatcccatgtttgcattttaatgactatccattcccatgaaaccagacaaccccaatattgctacattgtcttaattttaccaatttgacctttctAGttcccaaaggtaaccctggtctccctggacagccaggtcttataggacctcctggacttaaaggaaccatcggtgatatgggttttccagGTgagtgatgaaaatcttccaaatatttagtcccattaatgaaaggtggttcaatatctctttttttgtcagaaaagaggctggtgttgatagaatcagactgaaacgatatctgaggtaatcagtgggtagtgttc |
| **COL4A5-Exon36** | tcagtgggtagtgttctcttgttacacaaatatttaaaatgggaattaagggacaaaattgtagaaaccttaaaagttataattcacaaacatcttttctccagctatacccccttttatttaaacttcctttaaaagtaggcttttatataattgtgtttgtgaggctcattattatctaactcagagtttgcggagctttttaaaaatctttttgctttgtcatatgcatcttagataatccacaagtaaagcatattttgtaaaatattatatatcacatattttcaacAGggcctcagggtgtggaagggcctcctggaccttctggagttcctggacaacctggctccccaggattacctggacagaaaggcgacaaaggtgatcctggtatttcaagcattggtcttccaggtcttcctggtccaaagGTaatctttggcatatagttttaggcacatacttgagcagatatgaaattttttaatactatgctcattcctaagttttcattaaacaaactatagaatgacatagtatattctggataagagatttattccttttcttttctcttagatgataagaagatatgttttagggtttgtggc |
| **COL4A5-Exon36 with c.3107G>T** | tcagtgggtagtgttctcttgttacacaaatatttaaaatgggaattaagggacaaaattgtagaaaccttaaaagttataattcacaaacatcttttctccagctatacccccttttatttaaacttcctttaaaagtaggcttttatataattgtgtttgtgaggctcattattatctaactcagagtttgcggagctttttaaaaatctttttgctttgtcatatgcatcttagataatccacaagtaaagcatattttgtaaaatattatatatcacatattttcaacAGtgcctcagggtgtggaagggcctcctggaccttctggagttcctggacaacctggctccccaggattacctggacagaaaggcgacaaaggtgatcctggtatttcaagcattggtcttccaggtcttcctggtccaaagGTaatctttggcatatagttttaggcacatacttgagcagatatgaaattttttaatactatgctcattcctaagttttcattaaacaaactatagaatgacatagtatattctggataagagatttattccttttcttttctcttagatgataagaagatatgttttagggtttgtggc |
| **COL4A5-Exon36 with c.3181C>T** | tcagtgggtagtgttctcttgttacacaaatatttaaaatgggaattaagggacaaaattgtagaaaccttaaaagttataattcacaaacatcttttctccagctatacccccttttatttaaacttcctttaaaagtaggcttttatataattgtgtttgtgaggctcattattatctaactcagagtttgcggagctttttaaaaatctttttgctttgtcatatgcatcttagataatccacaagtaaagcatattttgtaaaatattatatatcacatattttcaacAGggcctcagggtgtggaagggcctcctggaccttctggagttcctggacaacctggctccccaggattacctggatagaaaggcgacaaaggtgatcctggtatttcaagcattggtcttccaggtcttcctggtccaaagGTaatctttggcatatagttttaggcacatacttgagcagatatgaaattttttaatactatgctcattcctaagttttcattaaacaaactatagaatgacatagtatattctggataagagatttattccttttcttttctcttagatgataagaagatatgttttagggtttgtggc |
| **COL4A5-Exon39** | attctccccaccacccttcgcaccttactctcttagatattctgaaactatattaaaatgaagaactgaaaggttttagatgctgaatgactattccttattttcattatcctccttcatatttttataacattttgtgatccaaaggagtgtctcaaaagcaccttgtttcttttggataaagaagggagcatatggaagtaaaagggagttggaaattggaaaactgggtgtaacctgctgtactcaattttttAGgtggtggaggtcatcctgggcaaccagggcctccaggcgaaaaaggcaaacccggtcaagatggtattcctggaccagctggacagaagggtgaaccagGTgctgtagtttttcatttttcctatttttctaattttctctgtgttgaatttaacttgcctttttattacccacagtgaaattgtatcttctttcttactaagctaccacacactttccccttttccta |
| **COL4A5-Exon39 with c.3538C>T** | attctccccaccacccttcgcaccttactctcttagatattctgaaactatattaaaatgaagaactgaaaggttttagatgctgaatgactattccttattttcattatcctccttcatatttttataacattttgtgatccaaaggagtgtctcaaaagcaccttgtttcttttggataaagaagggagcatatggaagtaaaagggagttggaaattggaaaactgggtgtaacctgctgtactcaattttttAGgtggtggaggtcatcctgggcaaccagggcctccaggcgaaaaaggcaaacccggtcaagatggtattcctggaccagctggatagaagggtgaaccagGTgctgtagtttttcatttttcctatttttctaattttctctgtgttgaatttaacttgcctttttattacccacagtgaaattgtatcttctttcttactaagctaccacacactttccccttttccta |
| **COL4A5-Exon41** | tttgggatttggtaggagatagaaatgactcttgtgaattccattaattgccctaatgtatgtgaatagctaaccttataagcaagcttgtaactcggtattatttatcttctaattatactttactttcatAGgccaaaagggtgatggaggattacctgggattccaggaaatcctggccttccaggtccaaagggcgaaccaggctttcacggtttccctggtgtgcagggtcccccaggccctcctggttctccgggtccagctctggaaggacctaaaggcaaccctgggccccaaggtcctcctgggagaccagGTtatgtccgtgagtggtaggagaatggtctatttattagtccatgtatttcgttttgctggcaggttattcagtctttaagactttagaatttttccggtgcattggaggatgttaaaaaaaagactttaaaatttgtgatataacttcttacaagtaaatagcttggttcatagctaactccatctatttccat |
| **COL4A5-Exon41 with c.3700C>T** | tttgggatttggtaggagatagaaatgactcttgtgaattccattaattgccctaatgtatgtgaatagctaaccttataagcaagcttgtaactcggtattatttatcttctaattatactttactttcatAGgccaaaagggtgatggaggattacctgggattccaggaaatcctggccttccaggtccaaagggcgaaccaggctttcacggtttccctggtgtgtagggtcccccaggccctcctggttctccgggtccagctctggaaggacctaaaggcaaccctgggccccaaggtcctcctgggagaccagGTtatgtccgtgagtggtaggagaatggtctatttattagtccatgtatttcgttttgctggcaggttattcagtctttaagactttagaatttttccggtgcattggaggatgttaaaaaaaagactttaaaatttgtgatataacttcttacaagtaaatagcttggttcatagctaactccatctatttccat |

Black letter: sequences of flanking introns; Green letter: sequences of target exons; Red letter: mutation sites; “AG”: sequences of acceptor splice sites; “GT”: sequences of donor splicing sites.

**Figure S1** Sequencing diagrams of variants with altered splicing in COL4A5


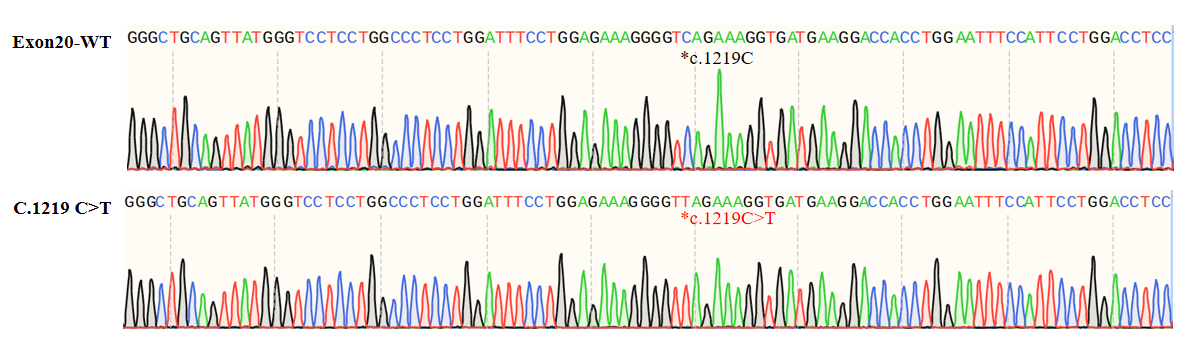

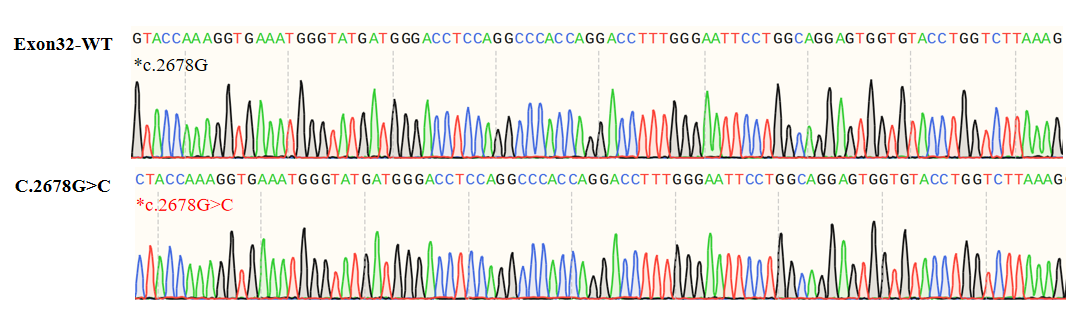

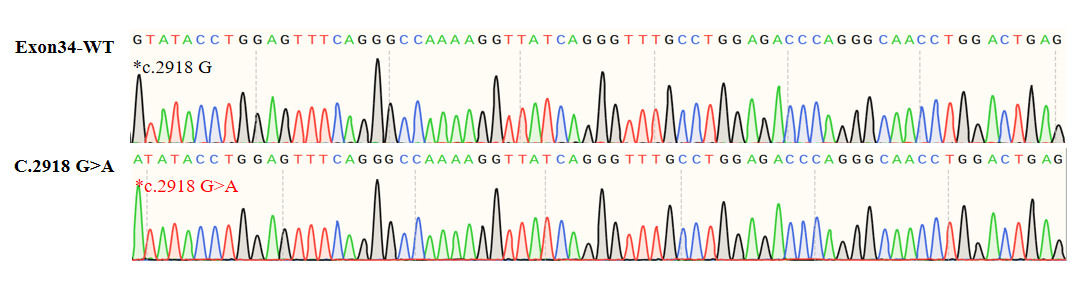

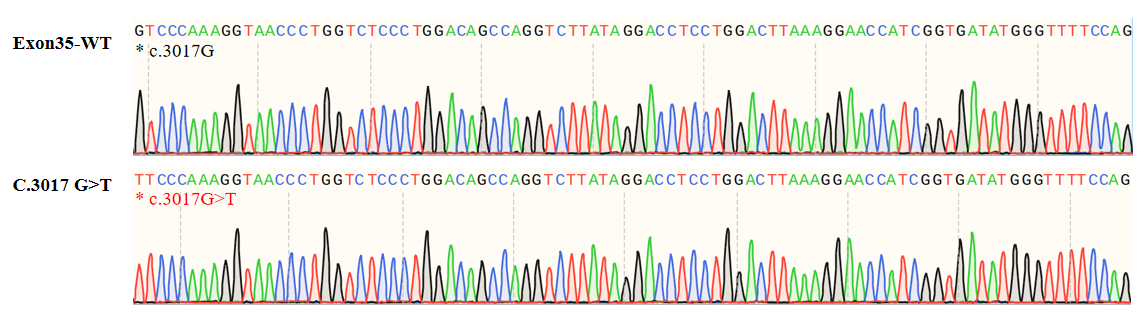

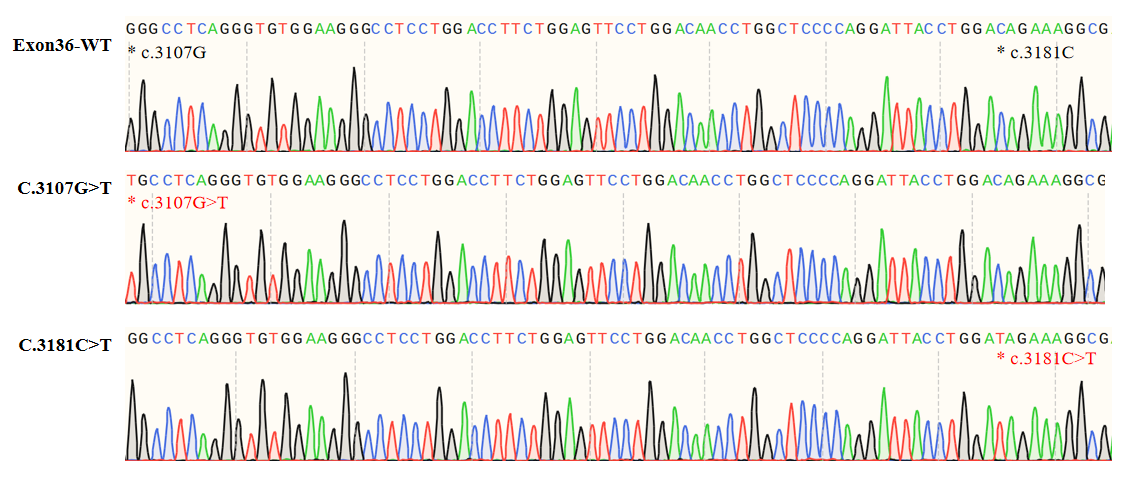

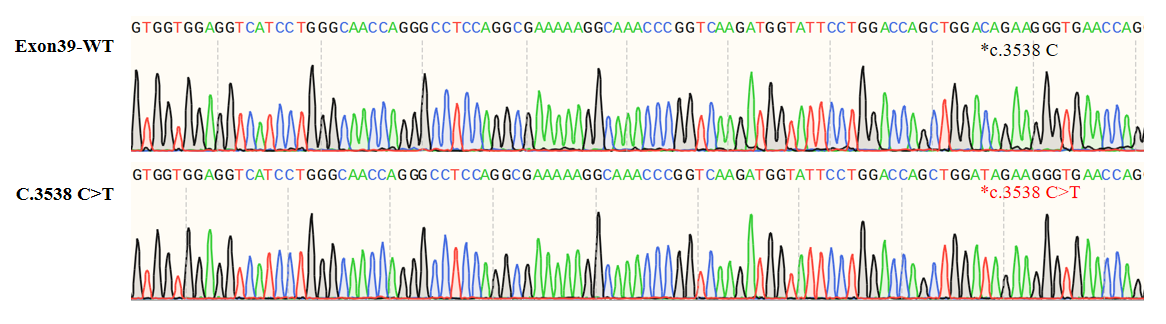

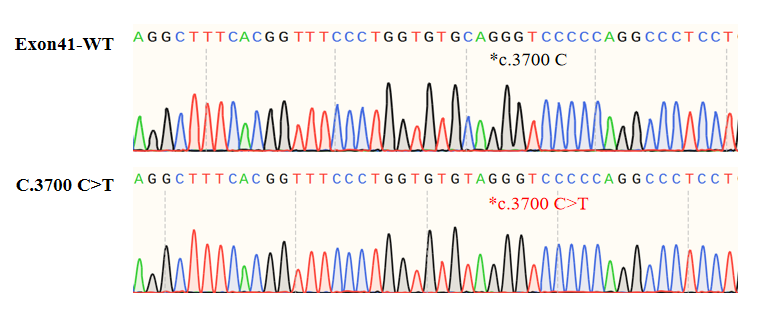

Supplement: Supplementary file 1 — Data S1. [file MGG3-12-e2395-s001.doc]
